# Dual identities for various alcohols in two different yeasts

**DOI:** 10.1080/21501203.2020.1837976

**Published:** 2020-12-07

**Authors:** Nitin Mahendra Chauhan, S. Mohan Karuppayil

**Affiliations:** a College of Natural and Computational Sciences, Dilla University, Dilla, Ethiopia; b Professor and Head, Department of Stem Cell & Regenerative Medicine and Medical Biotechnology, D Y Patil Education Society, Kasaba Bawada, Kolhapur, Maharashtra 416006, India

**Keywords:** Quorum sensing, *Candida albicans*, *saccharomyces cerevisiae*, biofilms, fusel alcohols, alcohol dehydrogenase

## Abstract

Most of the yeast bypasses the developmental stage from simple unicellular yeast to elongated structure like hyphae. Regulation of this transition is governed by various quorum sensing and signalling molecules produced under different conditions of growth, that differ significantly, both physiologically and chemically. The evidence of fungal quorum sensing was uncovered ten years ago after the discovery of farnesol as first eukaryotic quorum sensing molecules in Candida albicans. In addition to farnesol, tyrosol was identified as second quorum sensing molecules in C. albicans controlling physiological activities. After the discovery of farnesol and tyrosol, regulation of morphogenesis through the production of chemical signalling molecules such as isoamyl alcohol, 2-phenylethyl alcohol, 1-dodecanol, E-nerolidol, etc. is reported in C. albicans. Some of the evidence suggests that the budding yeast Saccharomyces cerevisiae exhibits this type of regulation and the signals are regulated by aromatic alcohols which are the end product of amino acid metabolism. The effects of these molecules on morphogenesis are not similar in both yeasts, making comparisons hard. It is hypothesized that these signals works in microorganisms to derive a competitive advantage. Here, we present an example for utilization of competitive strategy by C. albicans and S. cerevisiae over other microorganisms.

## Introduction

**Figure 1.**
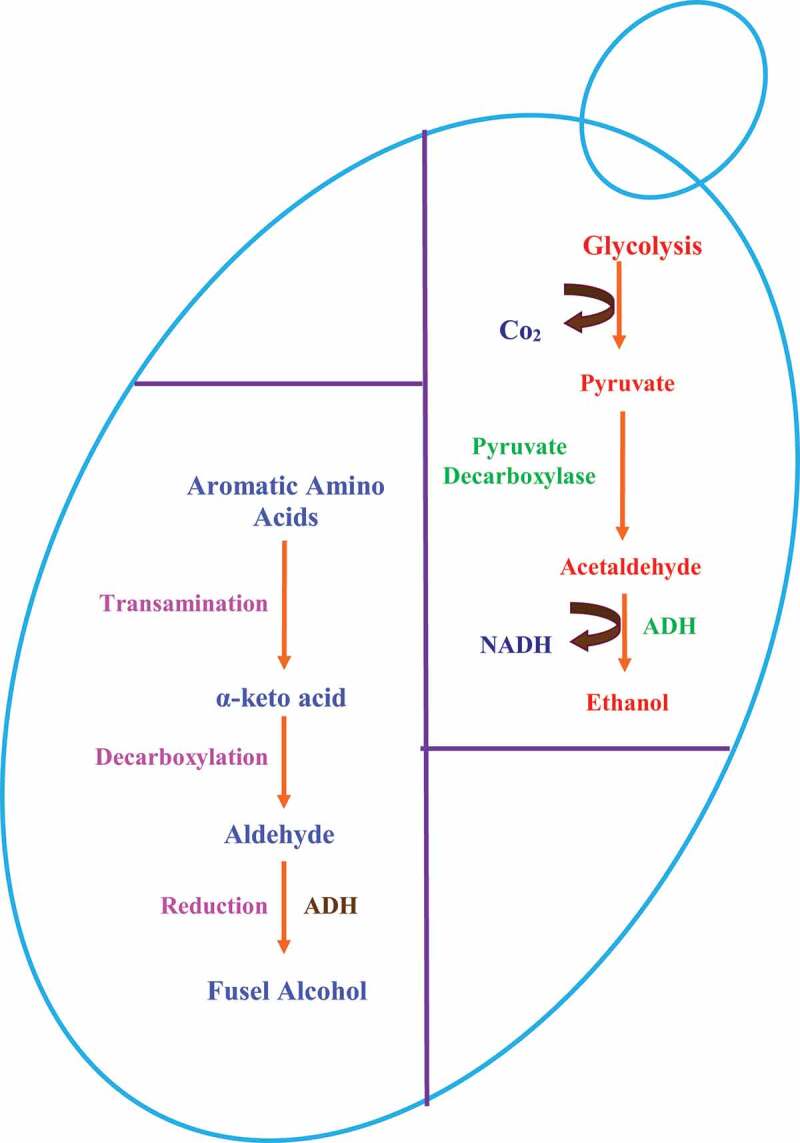
Regulation of alcohol production in yeast cells. The two common pathways observed in yeast cells are glycolysis and fusel alcohol production. Glycolysis is the major pathway in yeast cells that give rise to production of common metabolite, i.e. ethyl alcohol. Another pathway which generates different aromatic alcohols depends on various environmental factors, such as the availability of aromatic amino acids, the presence of ammonia, and an alkaline pH. Both pathways principally work under anaerobic conditions. The key element in above-mentioned pathway is alcohol dehydrogenase (ADH) which is reported to regulate downstream during morphogenesis and biofilm development

**Figure 2.**
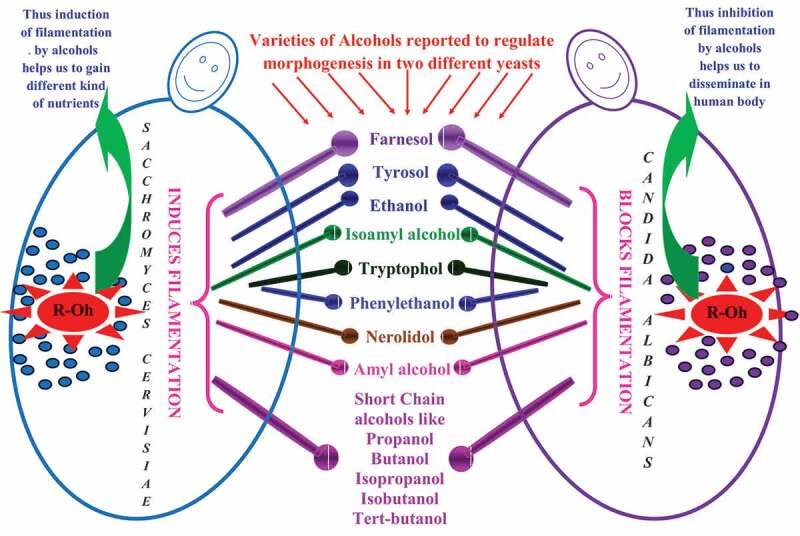
Differential behaviour for various alcohols in baker yeast *Saccharomyces cerevisiae* and human fungal pathogen *Candida albicans*. The effects of these molecules on morphogenesis are not similar in both the yeasts even though the mechanism of production is similar. For example in *Saccharomyces cerevisiae*, alcohols are reported to induce filamentation which is considered as a foraging response happens under nutrient poor conditions. However, in *Candida albicans* most of the alcohols produce opposite results that offer disseminative mode of growth which is crucial to establish infections. Apart from these, common advantage share by both yeasts is that alcohol production may offer an ecological advantage, since it could inhibit growth of other competing microorganisms under mixed-species population

Various environmental as well as nutritional factors are known to regulate morphogenesis in two widely studied yeast, i.e. *Saccharomyces cerivisiae* and *Candida albicans* (Saville et al. 2003; Biswas et al. 2007). It is under the regulation of quorum sensing molecules such as farnesol and tyrosol (Mosel et al. 2005; Chen and Fink 2006; Nickerson et al. 2006; Davis-Hanna et al. 2008; Dufour and Rao 2011; Barriuso et al. 2018; Padder et al. 2018; Mehmood et al. 2019). Different alcohols are produced by the yeasts *Saccharomyces cerevisiae* and *Candida albicans* (Ghosh et al. 2008; Hazelwood et al. 2008). For example, in presence of leucine, isoleucine, valine, phenylalanine, threonine, and tryptophan as nitrogen sources, both yeasts are reported to produce isoamyl alcohol, amyl alcohol, isobutanol, phenyl ethyl alcohol, propanol, isopropanol, and tryptophol (Thomson et al. 2005; Dickinson 2008; Hazelwood et al. 2008) (Figure 1). Alcohols such as ethyl alcohol, propanol, isopropanol, butanol, isobutanol, isoamyl alcohol, amyl alcohol, and tertiary-amyl alcohol are reported to induce filamentation in *S. cerevisiae* (Lorenz et al. 2000). *C. albicans*, in presence of tryptophol, phenyl ethyl alcohol, isoamyl alcohol, ethyl alcohol fail to switch from yeast to hyphae under standard induction conditions (Martins et al. 2007; Davis-Hanna et al. 2008; Chauhan 2009; Chauhan et al. 2011a).The effects of these molecules on morphogenesis are not similar in both the yeasts, making comparisons hard (Figure 2). Despite the tremendous literature about bacterial quorum sensing over the last few decades, quorum sensing in a eukaryotic organism is not clearly known until the discovery of farnesol as first eukaryotic quorum molecules (Hornby et al. 2001). However, over the last few years of hard work has changed the situation, which leads to more and more Pubmed articles with the keywords like *Candida* and farnesol; *Candida* and quorum sensing and too many. Apart from farnesol, tyrosol was second quorum sensing molecules discussed in eukaryotes, even though both were reported as autoinducer molecules in *C. albicans* around the 1970s (Lingappa et al. 1969). Although the fungal quorum sensing is still in its new stage which has changed our ideas and thus inhibiting these systems may present a novel approach to the development of new antifungal therapeutics. In addition to above two molecules, the other known fungal quorum sensing molecules are all alcohols derived from aromatic derived from amino acids tyrosine (tyrosol), phenylalanine (phenylethyl alcohol) and tryptophan (tryptophol) (Chen et al. 2004). Later these molecules were found to be the part of *S. cerevisiae* quorum sensing system (Alem et al. 2006). The purpose of this review is to strengthen the differential biological behaviour for various alcohols on *S. cerevisiae* and *C. albicans*, followed by other molecules which have not been identified yet.

## Regulation of filamentation by alcohols in baker yeast *Saccharomyces cerivisiae*

*S. cerevisiae* has a long history of use dating back to the last eight millennium in the production of alcoholic beverages. Along with ethyl alcohol and carbon dioxide, fermenting cultures of this yeast produces many low molecular weight compounds as by-products. These include alcohols, aldehydes, organic acids, esters, organic sulphides and various carbonyl compounds. During fermentation, *S. cerevisiae* produces various aliphatic and aromatic alcohols referred to as fusel alcohols (Hazelwood et al. 2008). Fusel alcohols are considered as by-products of organic compound dissimilation. Amino acids are the major sources of nitrogen in wort and grapes and these amino acids are taken up by the yeast. Amino acids that are widely utilised by yeast during fermentation are valine, leucine, isoleucine, methionine and phenylalanine (Hazelwood et al. 2008). Fusel alcohols are reported to be produced under aerobic as well as anaerobic conditions. The concentrations of aromatic alcohol produced under anaerobic conditions are slightly more than those under aerobic conditions (Hazelwood et al. 2008).

In addition to typical single-celled yeast morphology, *S. cerevisiae* forms elongated cells such as hyphae, pseudohyphae or invasive filaments in nature and *in vitro* conditions. The filament formation in baker yeast is considered as a foraging response happens under nutrient-poor conditions. Various fusel alcohols are reported to induce filament formation in nutrient rich as well as nutrient-poor conditions. Fusel alcohols, such as, 3- methyl-1- butanol (isoamyl alcohol), 2- methyl- 1- butanol (active amyl alcohol), 2- methyl- 1- propanol (isobutyl alcohol), 2- phenylethyl alcohol and 3- (2- hydroxyethyl) indole (tryptophol) which are the end products of leucine, isoleucine, valine, phenylalanine and tryptophan catabolism, respectively, are known be to produced by baker’s yeast (Dickinson 2000, 2008).

### Isoamyl alcohol as an inducer of morphogenesis

Induction of filamentation by isoamyl alcohol in *S. cerevisiae* was extensively studied by various co-workers. Martinez-Anaya et al. (2003), reported the induction of pseudohyphal phenotype by isoamyl alcohol results from the operation of the morphogenesis checkpoint. Isoamyl alcohol has been shown to delay in the nuclear division, which results in continuous budding. The whole process requires the activity of *Swe 1* gene. The *Swe 1* gene mainly functions as a protein kinase which is required for apical growth. *Swe 1* caused phosphorylation of *cdc 28. Cdc 28* is a structural protein required for septin formation. Such phosphorylation of *cdc 28* would be expected to delay in mitosis. The results of FACS analysis of isoamyl alcohol-treated cells showed an increase in the number of cells in G2 phase compared to that of untreated cells; indicating that mitosis was delayed. Later in 2007, Dickinson and his group (2008, p. 2009) reported transcriptome analysis of isoamyl alcohol-induced filamentation in *S. cerevisiae*. They found that *DLD 3* and *GRE 2* genes played important roles in filamentation. These two proteins codes for D-lactose dehydrogenase and methyglyoxal reductase. Mutants carrying *dld 3* mutations displayed reduced filamentation in 0.5% isoamyl alcohol and need a higher concentration of isoamyl alcohol to affect more complete filamentation. Hence, *DLD 3* seems to be required for a full response to isoamyl alcohol. Mutants carrying *gre 2* mutation were depressed for filament formation and forms large, invasive filament in absence of isoamyl alcohol. Therefore *gre 2* acts as a suppressor of filament formation by virtue of encoding isovalredehyde reductase (Lorenz et al. 2000).

Diploid cells of budding yeast *S. cerevisiae* when starved for nitrogen are reported switch to filamentous growth. Poor carbon sources like starches may stimulate filamentation. Isoamyl alcohol and butanol induce filament growth in haploid cells. Butanol also induces cell elongation and changes in budding pattern, which leads to pseudohyphal growth in liquid and solid medium. La Valle and Wittenberg (2001) showed that *Swe 1* is required for the production of pseudohyphae in response to 1-butanol. They found that *Swe 1* mutant do not develop pseudohyphae in response to 1-butanol. Another study showed that mutation in *STE 12* and *TEC 1* blocked butanol induced filamentation in *S. cerevisiae. STE 12* and *TEC 1* are the transcription factor which regulates *FLO 11* transcription downstream of the MAPK pathway. *FLO 11* is a cell surface flocculin which promotes agar invasion and cell adhesion in *S. cerevisiae* (Jiang and Kang 2003).

### Ethyl alcohol initiate filamentation

Ethyl alcohol is reported to stimulate filamentation of diploid cells under nutrient limiting conditions. *S. cerevisiae* mutant in *STE 12* and *TEC 1* blocked ethyl alcohol-induced filamentation. In contrast, ethyl alcohol suppressed filamentation defects *mep2/mep2, gpa2/gpa2* and *gpr1/gpr1* strains. *Mep 2, gpa 2* and *gpr 1* are the transcription factors. These proteins are the part of cAMP-PKA pathways that regulates filamentous growth in *S. cerevisiae*. They may function by activating adenylate cyclase and PKA pathway. Besides these other alcohols such as methyl alcohol, propanol, isopropanol, isobutanol, tertamyl alcohols are reported to induce filamentation in *S. cerevisiae*. However, the mechanisms of induction of filamentation by these alcohols are still need to be found out (Lorenz et al. 2000).

*S. cerevisiae* has been extensively studied for alcohol fermentation. During fermentation under aerobic and anaerobic, yeast recycles NADH in the acetaldehyde-to-ethyl alcohol. The conversion of acetaldehyde to ethyl alcohol is regulated by alcohol dehydrogenase. This enzyme catalyses the reactions in both directions, i.e. acetaldehyde to ethyl alcohol and from ethyl alcohol to acetaldehyde with different catalytic efficiencies (Piskur et al. 2006). *S. cerevisiae* encodes 7 ADH genes. The details of the role of alcohol dehydrogenase are given in Table 1.

**Table 1.** Role of alcohol dehydrogenase in two different yeasts (Source: Thomson et al. 2005)

Types of alcohol dehydrogenase		Functions	
		*S. cerevisiae*	*C. albicans*
*ADH 1*	*ADH 1*	Major enzyme responsible for the convesion of acetaldehyde to ethyl alcohol. *ADH 1* transcription is repressed when cells are grown on a non-fermentable carbon source such as ethyl alcohol or glycerol.	80% homologous to *SC ADH 1*. Expressed in yeast form but not in hyphal form. Responsible for the conversion of Aldehyde to ethyl alcohol. Restricts *C. albicans* biofilm formation through ethyl alcohol based mechanism.
*ADH 2*	*ADH 2*	Conversion of ethyl alcohol to acetaldehyde. 89% homologous to *ADH 1* but functions opposite. It helps *S. cerevisiae* to use ethyl alcohol as a carbon source.	70% hmologous to *SC ADH 2*. soluble in hyphae. Expression is regulated upon white opaque switching. Helps in ethyl alcohol degradation; Fermentation.
*ADH 3*	*ADH 3*	Nuclear gene of the mitochondrial matrix. 80% homologous to *ADH 1*. Glucose represses *ADH 3* expression. Helps in maintaining ethyl alcohol acetaldehyde redox balance during anaerobic growth.	Expression is regulated upon white opaque switching. Induced by nitric oxide. Helps in ethyl alcohol degradation; Fermentation; Isoleucine, leucine, Phenylalanine, Valine degradation.
*ADH 4*	*ADH 4*	Dimeric protein require zinc for function. It is often expressed low levelin laboratory strain but highly expressed in brewing strain.	Protein not essential for viability. Transcription increased in an azole resistant strain overexpressing MDR 1 and cells when exposed to fluconazole. Not involved in fusel alcohol production.
*ADH 5*	*ADH 5*	Share 76%, 77% and 70% homology to *ADH 1, ADH 2, ADH 3* respectively. Not main enzyme responsible for conversion of acetaldehyde to ethyl alcohol. Expression is increased in presence of xylose.	Soluble in hyphae. Expression is regulated upon white opaque switching. Fluconazole induced; Antigenic during murine systemic infection; regulated by *Nrg 1p, Tup 1p*; Macrophage downregulated protein.
*ADH 6 and ADH 7*	*ADH 6 and ADH 7*	Recently identified. Helps in lignin degradation and synthesis of fusel alcohol.	Not identified.

### Role of phenylethyl alcohol and tryptophol

Chen and Fink (2006) reported stimulation of diploid pseudohyphal growth in *S. cerevisiae* by phenylethyl alcohol and tryptophol. On Solid Low-Ammonium Dextrose agar low concentrations (< 20 µM) of either phenylethyl alcohol or tryptophol stimulated filamentation. Interestingly, the addition of both alcohols gave greater stimulation of pseudohyphal growth than alone. Phenylethyl alcohol increased haploid invasive growth of *S. cerevisiae*, whereas, tryptophol failed to promote haploid growth in baker yeast. The induction of filamentation growth requires the function of *FLO 11. FLO 11* mainly functions as cell surface flocculin, which promotes agar invasion and adhesion in baker yeast. *S. cerevisiae FLO 11* mutants were completely defective in diploid pseudohyphal and haploid invasive growth and these defects could not be suppressed by the addition of these alcohols. Chen and Fink (2006) also studied the expression of *FLO 11* gene by quantitative RT-PCR. They found that the expression of the gene increased approximately two fold in presence of phenylethyl alcohol and tryptophol. Collectively these data suggest that the alcohols of metabolic origin may have a quorum sensing role for filamentous growth, and may link signalling and regulatory pathways that influence morphogenesis. Both alcohols, i.e. phenylethanol and tryptophol are reported to regulate morphogenesis under nitrogen starvation and act as quorum sensing molecules in *S. cerevisiae* (Albuquerque and Casadevall 2012). Another report suggests that the effect of this compound which is produced during aromatic amino acid metabolism on different wine yeast (Gonzalez et al. 2018). Recently, the response of environmental isolates of *S. cerevisiae* filamentous growth to phenylethyl alcohol and tryptophol filamentous is found to be generalised highly variable phenotype under natural populations. However, *S. cerevisiae* response to this quorum sensing molecules is surprisingly rare (Lenhart et al. 2019).

### Farnesol and tyrosol function in S. cerevisiae

Low levels of farnesol are produced by many types of yeast used for wine making as a volatile flavour compound. The yeast *S. cerevisiae* is also known to produce a low level of farnesol. *S. cerevisiae* erg 20 mutant is defective in farnesol pyrophosphate synthatase and excretes five prenyl alcohols such as sopentenol, dimethylallyl alcohol, linalool, geraniol and very low concentration of farnesol as compared to wild-type *S. cerevisiae*. Farnesol also induced the generation of reactive oxygen species by inhibition of the mitochondrial electron transport chain in *S. cerevisiae* through the involvement of phosphotidyl transferase (Nickerson et al. 2006). In *S. cerevisiae*, tyrosol has no detectable effect (Chen and Fink 2006).

### Behaviour of short-chain alcohols in S. cerevisiae

Ethyl alcohol the primary fermentation product in yeast and another alcohol butanol are also known to induce filamentation in *S. cerevisiae*. 1% of butanol and 1% of ethyl alcohol are known to induce pseudohyphal growth in *S. cerevisiae*. The elements of the MAPK pathway are involved in the induction of pseudohyphal growth in this yeast. The *STE 12* and *TEC 1* upstream elements of the MAPK pathway and a part of the nitrogen sensor are responsible for this induction. *Ste 12* and *Tec 1* block colony filamentation stimulated by butanol. In some *Ste 12* mutant strains grown in presence of butanol, a weak residual filamentation was observed, whereas Tec 1 mutants completely blocked ethyl alcohol-induced pseudohyphal growth in *S. cerevisiae*. Whereas ethyl alcohol suppressed the pseudohyphal defects of *Mep 2, Gpa 2* and *Gpr 1* strains of *S. cerevisiae* which are the regulator of the MAPK network (Lorenz et al. 2000).These data strongly support the role of various alcohols as morphogenetic signalling in *S. cerevisiae* (Dickinson 2008).

## Response of human fungal pathogen *Candida albicans* to different alcohols

The dimorphic fungal pathogen, *Candida albicans* can grow and colonise different parts of human bodies (Whiteway and Bachewich 2007). The pH of the oral cavity, stomach, gastrointestinal tract and blood varies according to diet, metabolism of other microflora and salivary flow (Bensen et al. 2004). *C. albicans* can grow in all of these pH conditions. Significantly, *C. albicans* can adapt to aerobic, anaerobic or hypoxic microenvironments. In the gastrointestinal tract, *C. albicans* lives under anaerobic conditions (Dumitru et al. 2004). Similarly, the interior part of biofilms also supports anaerobic lifestyle (Nickerson et al. 2006). The ability to adapt in all of these conditions is one of the most important attributes of various fungal pathogens (Nickerson et al. 2006). It was identified that 16 metabolic pathways involved in central carbon metabolism were significantly up-regulated when *C. albicans* switches from yeast to hyphal form, whilst 11 metabolic pathways are down-regulated when a hyphal form was regulated by specific quorum sensing molecules like farnesol, tyrosol, isoamyl alcohol, phenyl ethyl alcohol, etc (Uppuluri 2006). These alcohols are known to inhibit translation to govern morphogenesis in *C. albicans* (Egbe et al. 2015). Thus it is clear that *Candida* can produce different signalling molecules which are crucial to its pathogenesis. Various chemical signalling molecules that govern morphogenesis in *Candida* are discussed below.

### The first eukaryotic quorum sensing molecule farnesol

Farnesol is the first quorum sensing molecule discovered in a eukaryote (Hornby et al. 2001). *C. albicans* is reported to produce farnesol. It is produced by an alternative pathway from the sterol biosynthetic intermediate, i.e. farnesyl pyrophosphate by *C. albicans*. Chemically farnesol is, 1-hydroxy-3, 7, 11-trimethyl-2, 6, 10-dodecatriene, with a molecular formula of C_15_ H_26_ O having a molecular weight of 222.37. It is a component of many perfumes (Hornby et al. 2003; Hornby and Nickerson 2004; Nickerson et al. 2006; Cottier and Muhlschlegel 2012; Polke and Jacobsen 2017). Drugs that block sterol synthesis can elevate farnesol production. Farnesol is identified as a quorum sensing molecules by its ability to block morphogenetic switching in *C. albicans*. Farnesol is reported to be produced aerobically within a temperature range of 15–42°C. Farnesol was not detected during anaerobic growth. *C. albicans* mutant in filamentous growth are able to produce 6–19 times more farnesol than the wild-type cells (Hornby et al. 2001; Nickerson et al. 2006). Farnesol is known to block germ tube formation in *C. albicans* triggered by various standard inducers such as a serum, N-acetyl-D-glucosamine, proline, RPMI-1640 medium, etc. At 1 µM farnesol, a 50% reduction in filamentation is reported. Interestingly, the concentration of farnesol needed was the same for liquid and solid media indicating that inhibition was not influenced by agar (Kruppa 2008).The concentration of farnesol needed for hyphal inhibition was influenced dramatically by serum (Mosel et al. 2005). Serum increased the level of farnesol required to halt filamentation in a concentration-dependent manner, which can reach up to 150–250 µM in presence of 10–20% of serum. Farnesol did not have any effect on germ tube elongation. Experiments with farnesol have shown that it can block morphogenesis when added at any time up to 30 minutes after inoculation. While filamentation was not blocked when farnesol was added after 90 minutes (Mosel et al. 2005). *tup1/tup 1* and *nrg1*/*nrg 1* mutants are strictly filamentous and that cells remain in filamentous form even in presence of added farnesol. Farnesol induces the expression of *Tup 1* mRNA, while the expression of *Tup 1* regulated genes, i.e. *Hwp 1* and *Rbt* decreases. Interestingly, the timing of expression corresponds to the commitment point (Kebaara et al. 2008). Sato et al. (2004) suggest the involvement of MAPK cascade in farnesol-mediated morphogenesis. *C. albicans* mutants in *Cph 1* or *Hst 7* or *Cst 20*, genes produce hyphae in presence of farnesol. All of these genes are elements of the MAPK cascade in *C. albicans*. Davis-Hanna et al. (2008) found that *C. albicans* hypha formation stimulated by *Ras 1- cAMP- Efg 1*, is inhibited by farnesol. They have found that a *Candida* strain carrying a dominant active variant of *Ras 1*, which forms hyphae under non inducing conditions, grew as yeast form in presence of farnesol. A recent report suggested that *CYR1* and *PDE 2* are involved in the molecular mechanism of farnesol on antifungal resistant to *Candida* biofilms (Chen et al. 2018).

### Tyrosol as secondary quorum sensing molecule in C. albicans

2-(4-hydroxyphenol)-ethyl alcohol, i.e. tyrosol, a derivative of tyrosine was identified as a second quorum sensing molecule after the discovery of farnesol in *C. albicans* (Chen et al. 2004). Tyrosol is known to be produced by *C. albicans* during planktonic as well as biofilm forms of growth. Tyrosol is undetectable up to 5 hours of incubation, but after 10 hours, it can reach to the concentrations ranging from 0.09 to 1.28 µM and reaches the maximum of 11.6 ± 1.3 µM after 48 hours. Compared to planktonic forms, *Candida* biofilms produced significantly more tyrosol (50%). *C. albicans* mutant in *Efg 1* and *Cph 1* are also reported to produce tyrosol at 37°C. Addition of 1 mM concentration of farnesol inhibits biofilm formation by 33%, whereas tyrosol at the same concentration did not show any effect. Even though exogenous tyrosol appeared to have no effect on biofilms, Scanning Electron Microscopy show that tyrosol stimulate hypha production during the early stages (1–6 hours) of biofilm development. Thus, tyrosol induces germ tube formation and accelerates biofilm development in *C. albicans* (Chen et al. 2004). However, the mechanism behind the regulation of morphogenesis by tyrosol is unclear.

The physiological roles of two quorum sensing molecules, i.e. farnesol and tyrosol are likely to depend on their respective concentrations, required to induce or repress gene expression. Tyrosol promotes hyphal formation during the early stages of biofilm, while farnesol plays a critical role in the later stages of biofilm through inhibition of filamentation, which permits the dispersal of yeast cells to colonise new surfaces. Farnesol inhibits the formation of germ tubes when the culture reaches a higher cell density. Tyrosol, triggers cell growth and formation of germ tubes at low cell density (Sprague and Winans 2006; Kruppa 2008).

### Role of dodecanol and 2-dodecanol

*C. albicans* is reported to produce dodecanol (Ghosh et al. 2008). Dodecanol at a concentration of 200 µM inhibited RPMI-1640-induced yeast to hyphal morphogenesis by 98 ± 7%. About 200 µM concentration of dodecanol did not cause any adverse effect on the growth of *C. albicans* cells, suggesting signalling roles for dodecanol. Davis-Hanna et al. (2008) reported for the first time, the involvement *C. albicans Ras 1- cAMP- Efg 1* signalling pathway in morphogenetic regulation by dodecanol. Hyphal growth induced by *Ras 1- cdc35- pka- Efg 1* cascade is repressed by dodecanol. However the addition of cAMP exogenously restored hypha and pseudohypha formation in cultures that contain dodecanol and this filamentation is dependent on *Efg 1* transcription factor because the rescue does not happen in the *efg 1/efg 1* background strain. *C. albicans* strain carrying active *Ras 1* variant which grew as a hyphae in the absence of inducers, grew in yeast form when the culture was supplemented with dodecanol (Martins et al. 2007; Davis-Hanna et al. 2008).

2-dodecanol is known to inhibit filamentation in *C. albicans* at a concentration of 0.005% (v/v). A study by Siew-Yang Lim et al. (2009) showed the involvement of *C. albicans SIR 2* gene in 2-dodecanol mediated morphogenesis. *Sir 2* protein is essential for white-opaque phenotype switching. However, *Sir 2* expression apparently correlated to cellular morphology, since low *Sir 2* expression was found in yeast form population of *C. albicans. Sir 2* functions as an upstream regulator of *Ras 1* gene and the addition of 2-dodecanol blocks *Sir 2* up-regulation under hyphal inducing conditions (Biswas et al. 2007; Siew-Yang Lim et al. 2009).

### Function of isoamyl alcohol in C. albicans

Isoamyl alcohol is a fusel alcohol derived from the amino acid leucine and is reported to be produced by *C. albicans* (Martins et al. 2007). Both planktonic and biofilms produce isoamyl alcohol. Planktonic form cells when cultured for 24, 48, 72, and 96 hours produce 64.09, 58.23, 59.36, and 57.91 µmol/g [dry/wt] of isoamyl alcohol, respectively. However, the 24 hours biofilm culture releases less isoamyl alcohol than those cultured for longer periods. *Candida* biofilm when grown at 24, 48, 72, and 96 hours releases 12.94, 45.17, 72.49, and 46.12 (µmol/g [dry/wt] of cells) of isoamyl alcohol, respectively. About 46 mM concentration of isoamyl alcohol inhibited cell growth by 80%. At 46 mM and 23 mM concentration of this compound, *Candida* filamentation was inhibited. 23 mM concentration isoamyl alcohol did not cause any effect on *C. albicans* cells. As isoamyl concentration goes on decreasing, the percentage of the hyphal formation increases diagonally (Martins et al. 2007; Ghosh 2009). The regulation of morphogenesis by isoamyl alcohol in *C. albicans* is reported by Egbe et al. (2015). They observed that the regulation of morphogenesis by isoamyl alcohol involves eukaryotic translation initiation factor eIF2B. However in the baker’s yeast *S. cerevisiae*, isoamyl alcohol produces opposite results, which results in the induction of filamentation, results from the operation of a morphogenesis checkpoint (Martinez-Anaya et al. 2003).

### Role of nerolidol, 2-phenylethyl alcohol and tryptophol

*C. albicans* planktonic and biofilm cells produce nerolidol *in vitro* (Martins et al. 2007). The amounts of nerolidol in *C. albicans* planktonic supernatants were almost constant from 48 to 96 hours of incubation, i.e. 2.42 to 1.96 (nmol/g [dry/wt] of cells). However, in *Candida* biofilms the picture was not the same, which showed 7.53 (nmol/g [dry/wt] of cells) of nerolidol when cultured for 72 hours. RPMI-1640 medium when supplemented with 1.5 µM of nerolidol caused a reduction in filamentation by 65–70%, without growth inhibition. The mechanism for nerolidol mediated inhibition of morphogenesis needs to be investigated (Martins et al. 2007; Ghosh 2009; Tati 2010).

The release of 2-phenylethyl alcohol by planktonic and biofilm cells, increased during 24–96 hours of growth. Planktonic supernatant produced 7.37, 18.44, 24.62 and 43.22 (µmol/g [dry/wt] of cells) at 24, 48, 72 and 96 hours of growth, respectively. The biofilm form of cells released 5.29, 29.39, 37.12 and 88.77 µmol/g [dry/wt] of cells of 2-phenylethyl alcohol at 24, 48, 72 and 96 hours of biofilm development. About 500 µM of 2-phenylethyl alcohol did not affect the growth of *C. albicans*, but inhibited yeast to hyphal transitions by 95% (Martins et al. 2007; Ghosh et al. 2008). The mechanism behind the inhibition of morphogenesis by 2-phenylethyl alcohol is not yet known.

High concentrations of tryptophol, i.e. >500 µM reduced filamentation in *C. albicans*. The inhibition was not due to the inhibition of *Candida* growth, because at 500 µM to 500 mM, tryptophol did not dramatically affect the growth of *Candida* cells. Tryptophol is also reported to affect *Candida* biofilms. But, the concentrations required are relatively high than what is required for inhibition of filamentation in human fungal pathogen *C. albicans* (Martins et al. 2007; Ghosh et al. 2009). Mechanism of modulation of morphogenesis by tryptophol is needed to be addressed.

### Response of *C. albicans* cells to ethyl alcohol and acetaldehyde

Even though ethyl alcohol is known to be produced by *C. albicans*, its physiological roles are not well studied. Previous data from our lab demonstrated the effect of ethyl alcohol on induced morphogenesis and biofilm development. About 4% of ethyl alcohol completely halted filamentation without affecting the growth and viability of *C. albicans* yeast phase cells. Ethyl alcohol also inhibited elongation of germ tubes. *Candida* biofilms development in presence of 4% ethyl alcohol was inhibited and a layer of adhered yeast cells was predominant and noted (Chauhan 2009). Based on this result we had already proposed a potential morphogenetic regulatory role for ethyl alcohol in *C. albicans* for the first time (Chauhan et al. 2011a).

We have administrated a scientific study on the *in vitro* effects of ethyl alcohol on induced ontogeny in *C. albicans*. Ethyl alcohol blocked hyphal form morphogenesis in planktonic forms at various time points studied. The effect was hypha specific and the inhibitory concentrations did not alter yeast phase growth and viability of *C. albicans* at various time points. Scanning electron microscopy pictures of *Candida* biofilms in presence of ethyl alcohol demonstrated that 4% of ethyl alcohol inhibited biofilm development and patches of cells predominantly consisting of yeast forms were observed. In the absence of ethyl alcohol, biofilms consisted of a network of yeast and filamentous cells. It is reported that growth inhibitory concentrations of ethyl alcohol produced a metabolite that relieves growth inhibition in the baker yeast *S. cerevisiae*. The growth inhibitory factor was identified as acetaldehyde. About 0.01% of acetaldehyde reduced susceptibility of yeast cells to ethyl alcohol. Furthermore, acetaldehyde concentration above 0.01%, no reduction in growth was observed (Walker-Caprioglio and Parks 1987). Interestingly, the same concentration of acetaldehyde (0.01%) did not relieve the toxicity of *C. albicans* cells to 10% of ethyl alcohol (Chauhan et al. 2011b).Glucose is one of the *in vitro* inducer of filamentation in *C. albicans*. However, the addition of various concentrations of glucose to medium containing serum and various concentrations of ethyl alcohol did not produce any reversible effect on morphogenesis. Based on this study, we propose a morphogenetic regulatory role for ethyl alcohol in *C. albicans* (Chauhan et al. 2011a).

Acetaldehyde is known to be formed in the human body by liver alcohol dehydrogenase. To a lesser extent, ethyl alcohol is oxidised to acetaldehyde by other tissues such as kidneys, respiratory tract, intestine and bone marrow (Jokelainen et al. 1994). *In vivo* colonic bacteria can significantly contribute to the production of acetaldehyde from alcohol (Jokelainen et al. 1994). Acetaldehyde is reported to be produced in saliva, through the metabolic activity of oral microorganisms (Tillonen et al. 1999). In alcoholics, acetaldehyde is believed to be produced from ethyl alcohol, predisposing them to cancer (Poschl and Seitz 2004). However, the potential effect of acetaldehyde on resident microflora of the human body is not investigated. *C. albicans* is known to produce acetaldehyde in the culture supernatants (Mukherjee et al. 2006). Mukherjee et al. (2006) has reported production of 0.01 mg/ml acetaldehyde by *C. albicans*. An *ADH 1* mutant produced up to 0.14 mg/ml of acetaldehyde. Interestingly, *C. albicans* isolated from high acetaldehyde-producing saliva produced more acetaldehyde from ethyl alcohol (Tillonen et al. 1999). Acetaldehyde was found to act as a substitute for xanthine oxidase-myeloperoxidase halide system that caused damage to *C. albicans* hyphae (Diamond et al. 1980). The effects of acetaldehyde on the physiology of *C. albicans* and particularly on morphogenesis are unclear. Our study, for the first time, show morphogenetic regulatory properties for acetaldehyde in *C. albicans*. It inhibited yeast to hyphal dimorphism induced by four standard inducers at 37°C, in a concentration-dependent manner. About 7 mM concentration of acetaldehyde which completely inhibited morphogenesis, did not adversely affect growth and viability at all time points studied (Chauhan et al. 2011b). However, above this effective concentration, viability decreased. Interestingly, 7 mM of acetaldehyde, prevented hyphal forms in the biofilm of *C. albicans*. Addition of 7 mM and 14 mM also inhibited (less than 50%) biofilm development significantly, and only adhered yeast cells were evident (Chauhan et al. 2011b). The mechanisms behind these effects are unclear. Aranda and Olmo (2004) have reported that *S. cerevisiae* when exposed to 1 mg/ml concentration of acetaldehyde produces adverse effects on cell cycle progression, DNA replication and protein biosynthesis, without significant effects on viability. Also, the induction of *S. cerevisiae MET* gene involved in sulphur metabolism and repression of genes involved in the growth and maintenance of cell polarity, was seen in response to acetaldehyde (Aranda and Olmo 2004). Similar responses may occur in *C. albicans. In vivo*, at locations where *C. albicans* predominates, accumulation of acetaldehyde may inhibit hyphal forms to favour yeast form cells. This may facilitate dissemination of *C. albicans* inside the host. Acetaldehyde mediated prevention of hyphal forms may inhibit typical biofilms but favour easily detachable “yeast only” biofilms. We propose morphogenesis inhibitory properties for acetaldehyde in planktonic as well as biofilm forms of *C. albicans*.

### Short-chain alcohols in C. albicans

We also studied for the first time the effect of pentanol, butanol, propanol, isobutanol, isopropanol and tertiary-butanol on morphogenetic switching, biofilm formation, growth and viability in *C. albicans* (Chauhan 2012). Among the various alcohols studied, pentanol (amyl alcohol) was found to be very effective. About 0.5% of pentanol completely blocked induced morphogenesis without affecting the growth and viability of *C. albicans*. Propanol and butanol inhibited morphogenesis completely at 1%. Isopropanol, isobutanol and tertiary-butanol produced the same effect at 2%. About 0.5% of pentanol caused a 50% reduction in metabolic activity in biofilms. While for other alcohols, hyphal inhibitory concentrations caused a 35–45% decrease in metabolic activity in *Candida* biofilms. Characterisations of biofilms in presence of alcohols revealed that 2% of butanol, isobutanol, tertiary-butanol, propanol and isopropanol inhibited filamentation in biofilms and favoured yeast dominated biofilms. Pentanol caused considerable inhibition of biofilm at 0.5% concentration leading to yeast-dominated biofilm formation (Chauhan et al. 2013).

## Conclusion and future perspective

In the natural environment, microbes exist in different conditions of growth, which significantly differ, both physiologically and chemically. The behaviour and production of metabolites by *C. albicans in vivo* may greatly vary depending on the niches it colonise in the human body, like the gastrointestinal tract, urogenital, or oral cavities (Ghosh et al. 2008). In deep-seated mycosis or in biofilms on prosthetic devices, it may live in a polymicrobial environment and may be subject to anaerobic conditions or exposed to the metabolites of its own or that of other organisms (Dumitru et al. 2004; Dufour and Rao 2011). In the gastrointestinal tract, deep-seated mycosis, and biofilms it may produce a considerable amount of alcohols, because of the anaerobic conditions. *C. albicans* is known to produce alcohols, such as dodecanol, ethyl alcohol, farnesol, tyrosol, isoamyl alcohol, nerolidol, etc. (Hornby et al. 2001; Alem et al. 2006; Chen and Fink 2006; Martins et al. 2007; Chauhan 2009). Morphogenesis in *C. albicans* is subject to the influence of various metabolites. For example, *C. albicans* yeast to hyphal form transition is inhibited by dodecanol, farnesol, isoamyl alcohol, nerolidol and phenyl ethyl alcohol (Chen and Fink 2006; Martins et al. 2007; Davis-Hanna et al. 2008). Interestingly, some of these metabolites induce filamentation in the model yeast, *Saccharomyces cerevisiae*, making distinguishable hard. The differential behaviour of yeasts suggests that fungi may have evolved signalling responses, which are species-specific that may be crucial for pathogenesis especially for *C. albicans* (Chen and Fink 2006).

**Figure 3.**
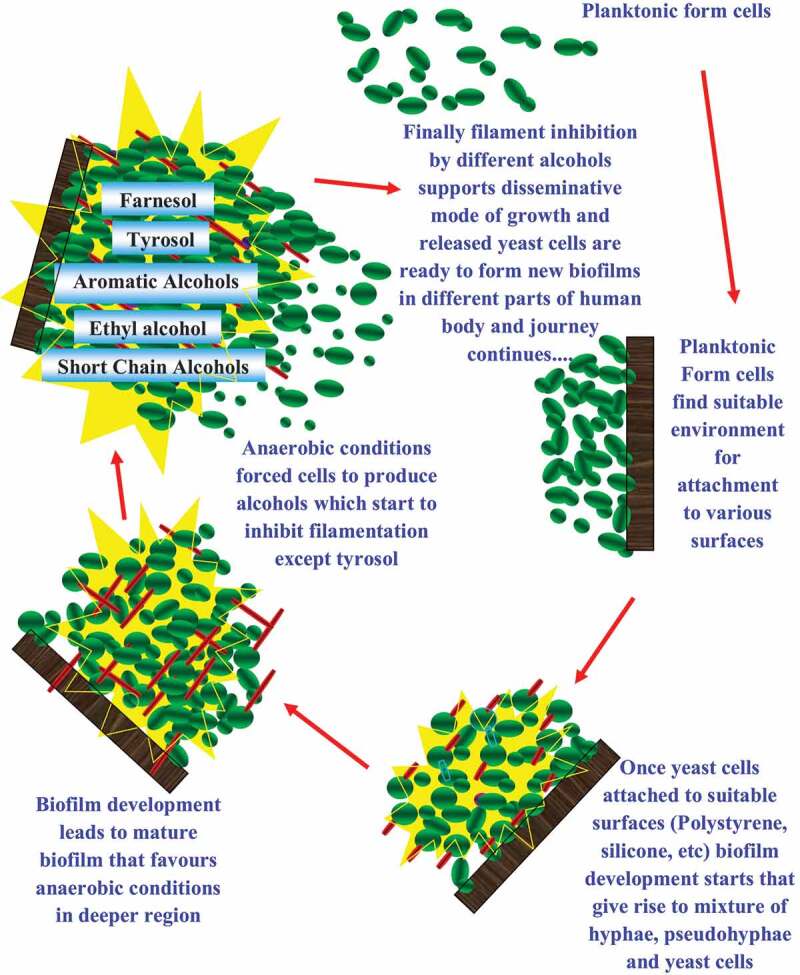
Rolling circle model for role of alcohols in biofilm of human fungal pathogen *Candida albicans. C. albicans* can form biofilms on various surfaces for e.g. Silicone, Polystyrene, etc. Deeper regions of biofilms support anaerobic conditions which favours the production of different alcohols. Thus alcohol production inhibits filamentation in biofilms and allows dissemination of yeast cells. Finally yeast cells released from biofilms are ready to form new biofilms in different parts of human bodies and journey continues

Our study showed that ethyl alcohol, isoamyl alcohol, propanol, butanol, isopropanol, isobutanol, tertiary butanol, pentanol and dodecanol inhibited filamentation in biofilms to give only yeast form biofilms which is different from the control that had a mixture of yeast and hyphal form cells. It is known that yeast only biofilms formed on a solid surface are easy to remove as compared to that of normal biofilms containing a mixture of yeast and hyphae. Under natural conditions *C. albicans* grow as a community, i.e. as a biofilm. The dense growth of biofilm may favour anaerobic conditions resulting in the production of alcohols. Production of alcohols may favour yeast phase cells causing release and dissemination of yeast cells from biofilms (Figure 3). Alcohol production *in vivo* may offer an ecological advantage, since it could inhibit the growth of other competing microorganisms (Piskur et al. 2006). *C. albicans* and *S. cerevisiae* which is relatively tolerant to alcohols may gain dominance over microorganisms that are susceptible to the alcohols. Inhibition of germ tube formation and elongation by ethyl alcohol may restrict the mobility of hyphae affecting tissue invasion and establishment of infection, *in vivo*. Results of this study suggest a potential morphogenetic regulatory role for these alcohols in planktonic as well as biofilm forms of *C. albicans*.

In the end, fusel alcohols are the natural products of amino acid catabolism. Both yeasts cannot use branched or aromatic amino acids as their sole carbon source. However they can be used as a nitrogen source under nitrogen limiting conditions, with the consequent production of fusel alcohols as a byproduct. The results described in the above review showed that aromatic alcohols are capable of stimulating the developmental transition from unicellular yeast to filamentous form. The findings that these alcohols exert different effects on morphogenesis in *S. cerevisiae* and *C. albicans* show that these molecules have a distinct species-specific effect (Table 2). Why alcohols inhibit morphogenesis and biofilm formation? Yeast cells can metabolise the available nutrients and amino acids as a nitrogen source. Availability of particular nitrogen source may increase the concentrations of respective alcohols. For example, in presence of leucine, isoleucine, valine, phenylalanine, threonine, and tryptophan as nitrogen sources, *S. cerevisiae* produces isoamyl alcohol, amyl alcohol, isobutanol, phenyl ethyl alcohol, propanol, isopropanol and tryptophol, respectively (Dickinson 2000; Lorenz et al. 2000; Hazelwood et al. 2008). Secondly, deeper regions of mature biofilms may produce anaerobic conditions. Anaerobic conditions may also persist in deep-seated mycosis (Dumitru et al. 2004). *C. albicans* may produce various levels of alcohols under anaerobic conditions of mature biofilms or under deep-seated mycoses. The type of alcohol produced may vary depending on the amino acid availability. The alcohols may exert effects depending on its concentrations. At lower concentrations (like 0.5% in case of amyl alcohol) it may inhibit morphogenetic switching and support yeast phase growth. At high concentrations, it slows down the growth rate. Most of the alcohols may exert a similar effect. Favouring of yeast phase growth that may favour disseminative mode of growth and also aid to escape from the alcohol-poisoned environment. It may also offer ecological advantages for *C. albicans* over other competing organisms which are more susceptible to alcohols than *Candida* in polymicrobial biofilms. It may be possible that instead of sensing the alcohols, cells may sense the intermediates during the conversion of amino acids to alcohols. Addition of alcohols to the medium may give rise to increased concentrations of respective intermediates. This possibility cannot be left out. We have demonstrated a morphogenetic role for acetaldehyde, the first oxidation product of ethyl alcohol metabolism on *Candida* dimorphism and biofilm formation. Although the production of these molecules is regulated similarly in both the organisms by environmental conditions, the differential response to these molecules in non-pathogenic *S. cerevisiae* and pathogenic *C. albicans* must reflect the fundamental difference between two organisms in sensing and processing these signals. The mechanism underlying behind morphogenesis regulation by these alcohols will give close up of biology of yeast to hyphal form transition and biofilm formation in *C. albicans* and *S. cerevisiae*. These provide a new insight in developing new antifungal targets especially against the pathogenic yeast *C. albicans* and *S. cerevisiae*.

**Table 2.** Morphogenetic properties of alcohols in two different yeasts

Alcohols	Production		Morphogenetic Role		References
	*S. cerevisiae*	*C. albicans*	*S. cerevisiae*	*C. albicans*	
Ethyl alcohol	Yes	Yes	Induction	Inhibition	15, 17, 18
Isoamyl alcohol	Yes	Yes	Induction	Inhibition	4, 16
Isobutyl alcohol	Yes	Not Known	Induction	Inhibition	15, 50, 57
Butyl alcohol	Yes	Not Known	Induction	Inhibition	15, 50, 57
Phenylethyl alcohol	Yes	Not Known	Induction	Inhibition	14, 16
Tryptophol	Yes	Yes	Induction	Inhibition	14, 16
Dodecanol	Yes	Yes	Induction	Inhibition	4, 16
Nerolidol	Tes	Yes	Induction	Inhibition	4, 16
Propanol	Yes	Not Known	Induction	Inhibition	15, 50, 57
Isopropanol	Yes	Not Known	Induction	Inhibition	15, 50, 57
Methyl alcohol	Yes	Not Known	Induction	Inhibition	15, 50, 57
